# Estimating the Marginal Effect of Socioeconomic Factors on the Demand of Specialty Drugs

**DOI:** 10.5539/gjhs.v7n2p28

**Published:** 2014-09-28

**Authors:** Seyede Sedighe Hosseini Jebeli, Mohsen Barouni, Parvane Heidari Orojloo, Sattar Mehraban

**Affiliations:** 1 Health Services Management Department, School of Public Health, Ahvaz Jundishapur University of Medical Sciences, Ahvaz, Iran; 2 Research Center for Health Services Management, Institute forFutures Studies in Health, Kerman University of Medical Sciences, Kerman, Iran; 3 Health Management and Economics Research Center, School of Health Management and Information Sciences, Iran University of medical sciences, Tehran, Iran; 4 Researcher in the Field of Health Economics, Golestan Province, Iran

**Keywords:** demand, specialty drug, probit model, Marginal effect

## Abstract

Given the growing importance and role of drugs in the treatment of diseases, as well as replacement of them rather than expensive and often unsafe procedures, study of socioeconomicfactors affecting future demand for them seems necessary. we seek to examine the extent of to which socioeconomic factors affect specialty medicine use by the patients.using data from questionnaires completed by 280 patients with multiple sclerosis, hemophilia, thalassemia, and chronic kidney disease, we estimate marginal effect of significant variables in probitmodel. We found that the need for the patient(ME=0.858), deterioration of the patient (ME=-0.001), household size (ME =0.0004), House Ownership (ME=-0.002), gender (ME=-0.04), income (ME=-0.0007), education (ME=-0.0021) and job (ME=-0.0021) are significant variables affecting demand for specialty drugs. We conclude that it can be programmed to promote and protect the welfare of patients by specific factors such as income, and largely affect the demand of medication and medical services. Therefore economic aid to these patients should not be limited only to medical subsidies, especially in patients with MS, income and welfare can reduce drug demand.

## 1. Introduction

Healthcare is one of the essential needs of the population, today, the various governments in the world, consider the appropriate health care as the most successful function of their services. In recent decades, due to the high costs of health care and problems of financing for most governments, Policymakers and politicians have accepted that health is not just a social issue and economic aspects should also be considered. The pharmaceutical industry plays an important role in the health economics (Jahanmehr, 2009). Today there are various drugs to treat a variety of ailments from the common cold to skin diseases and cancers and other incurable diseases (Sabaghkermani, 2006). The affordability of life-saving drugs is of critical importance in all countries that are afflicted by deadly diseases like AIDS. Unaffordable treatments in developing countries are a source of welfare losses and slowdown growth by making human capital obsolete. In developing countries 50 to 90 percent of drugs are paid out-of-pocket as a share of total health expenditures (Caldera & Zarnic, n.d.).

Costly diseases such as thalassemia, hemophilia, chronic renal failure, dialysis, MS, hepatitis, diabetes, AIDS, epilepsy, Parkinson’s, autism, are called special diseases. Unfortunately, right now many people are suffering from this disease. Due to the nature of specialty drugs that have a very high cost, and patient sometimes cannot find an alternative, In this study, we decided to investigate the factors affecting demand for specialty drugs.

In a study of (Curtis et al., 2004), an estimated 34 million elderly people filled 630 million prescriptions in 1997. Thirty-seven percent did not have prescription drug insurance. Total prescription drug expenditures exceeded $23 billion. Persons without prescription drug insurance spent slightly less than $7 billion; those with insurance spent more than $16 billion. After controlling for health status, comorbidity, and demographic characteristics, prescription drug insurance increased expenditures by $183 per person. The marginal increase in total expenditures of extending the average observed benefit to those currently uninsured is $2.3 billion (95% confidence interval, $1.2-3.5 billion).

In the study, (Leth-Petersen & Skipper, 2010) entitled “income and Use of prescription drugs for people nearing retirement” changes in the demand for drugs and income were assessed. The results of cross-sectional estimates have shown a strong relationship between income and the demand for prescription drugs.

In the study, (Qiasvand et al., 2009), Determinants of Catastrophic health care expenditures in Hospitals Affiliated to Iran University of Medical Sciences, among the 16 variables, 8 variables including gender, illness of family members, number of household members, number of hospitalizations, the level of household income and home ownership and coverage of health insurance, were significantly associated with the likelihood of exposure to catastrophic health care expenditures.

Several authoritative studies the effects of variables such as out of pocket payment, insurance, education, gender, income and the price on demand of drugs. In a study, (Stern & Riesman, 2006), the relationship between the out of pocket payments (OOP) and Continuity of MS treatment was examined. It was shown that the expenditures paid out of pockets for MS drugs more than $ 200 compared to pay out of pockets spending less than $ 100 has a 6 times more possibility to leave the treatment. In the study (Sari & Langenbrunner, 2001) carried out using binary regression model, the results showed that high-income groups paid more in absolute terms. But low-income groups, paid a greater proportion of their income for their drugs.

In a report titled “The demand for prescription drug as a tool for cost sharing RAND institute,1985”, drug demands in the different levels of cost sharing have been discussed. Participants in the trial, randomly place in different coinsurance and franchise groups. The findings show that people with more generous insurance benefits buy more prescription pharmaceuticals.

In the study, (Huttin, 2000), which is entitled “Cluster Analysis of drug Expenses and income elasticity changes” in America, an analysis of regional groups of individuals with differentsocio-economic profile only a small number of groups showed positive income elasticity.

## 2. Method & Material

This cross-sectional study was conducted in the context of the major centers and referral pharmacies selling specialty drugs; like the Red Cross, and Community Foundation for Special Diseases. The study population consisted of households that have at least one of their members suffer from the disease: thalassemia, hemophilia, chronic renal failure, dialysis treatment and MS. and referred to above centers.

Using correlation coefficient (r) for the income level and drug demand in previous studies and the formulation of George Norman (2003), with values below sample size were estimated 70 household. But since we checked in 4 groups of disease, to enhance the reliability of the research, 280 patients were included.


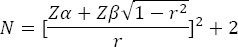


Zα=2.575; Zβ=1.282; r = 0.45α=0.05

Data collection tool was household budget questionnaire which belongs to Statistical Center of Iran and contains three sections: demographic information, economic characteristics and medical expenditures.

**Table 1:** Variables

Socio-economical factors	Socio-economical variables	Coding
Job	Governmental	Governmental=1 Non Gov& Private=0
	Non Governmental	Non Gove=1Gov&Private=0
	Private	Private=1Gov&Non Gov=0
Education	Illiterate	Illiterate=1High school&University=0
	High school	High school=1 Illiterate&University=0
	University degree	University=1 Illiterate&High school=0
Drugstores	Charities	Charities=1Gov–Private=0
	Governmental	Gov=1 Charities&Private=0
	Private	Private=1Charities&Gov=0
Disease Deterioration	Deterioration	Deterioration=1
	No Deterioration	No Deterioration=0
House Ownership	owner	Owner=1
	Tenant	Tenant=0
Recognizing the need	Recognizing the need	Recognizing the need=1
	No Recognition	No Recognition=0
Insurance coverage	Insurance coverage	Insurance coverage=1
	no Insurance coverage	no Insurance coverage=0
Sex	Men	Man =1
	Women	Woman=0
Continuous variables	Age	
	Income	
	Frequency of use	
	Household size	

### 2.1 Descriptive Statistics

According to the survey, 87.3% of household heads were male and the rest were women. Descriptive statistics also shows that 68.2% owned a house and 31.8% were tenants considered. The highest prevalence age group were 41–50 years (25.1%) and least frequent in the age group was observed in 21–30 (7.9%). Variable Frequency also shows that 77.15% of households have no insurance coverage. In relation to the type of pharmacy, government agencies, with 63.3% had the highest frequency.

**Table 2:** 

Sex		Recognizing the need		Insurance coverage		Disease Deterioration		House Ownership		

Percent	frequency	Percent	frequency	Percent	frequency	Percent	frequency	Percent	frequency	Reply
12.73	34man	15	252	206	61	61.8	165	68.16	182	yes	
87.28	233woman	5.62	94.33	77.15	22.85	38.2	102	31.84	85	no	

Source: research findings.

**Table 3:** 

Job			Drugstores			Education level		

Percent	frequency	Description	Percent	frequency	Description	Percent	frequency	Description
27.34	73	Governmental	7.12	19	Charities	9.74	26	Illiterate
44.57	119	Non Governmental	63.3	169	Governmental	67.04	179	High school
28.09	75	Private	29.59	79	Private	23.22	62	University degree

Source: research findings.

**Table 4:** 

Variables	Mean	SD	Min	Max
Household size	3.94382	1.663583	1	10
Age)head of Household)	49.11985	13.59375	21	86
Income	1.64e+08	4.65e+08	0	4.23e+09
Frequency of use	33.22472	50.12603	1	360

Source: research findings.

### 2.2 Econometrics Model

Economists usually assume that the dependent variable is continuous set of values. There is, however, numerous cases of decision-making behavior that can be summarized in the form of a limited set. Models which are used for such purposes are called models with qualitative dependent variables. Simplest of these models are models in which the dependent variables are binary. For the dependent variable, there are only two values, zero and one. For instance, a person could buy drugs or to unsubscribe. Statistical models used in this study is the Logit and probit. Formation of many discrete choice models is based on economic random utility theory. These models are based on utility maximization for each item. Depending on the probability density function for the error term, Type of discrete choice model will specified. If we assume a normal distribution, the difference will be normally distributed. The normal cumulative distribution function, gives the probit model. Probit probabilities are obtained by solving the following integral:


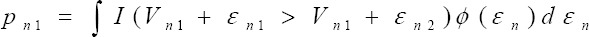


This equation φ represents the normal distribution cumulative standard, and is utility option.

If the non-visible part of the Gumbel distribution is assumed, the difference between the logistic distribution gives the binary Logit model. Due to the closed form integral Logit model, the probability of binary Logit model becomes the following simple relationship:


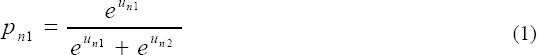



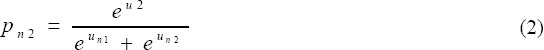



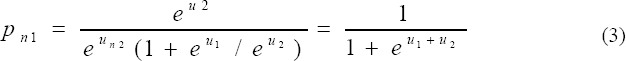


In the Logit model, errors are assumed to follow the standard logisticdistribution with mean 0 and variance 
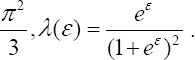
. The errors of probit model areassumed to follow the standard normal distribution, 
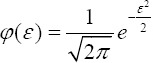
 with variance 1.

**Figure 1:**
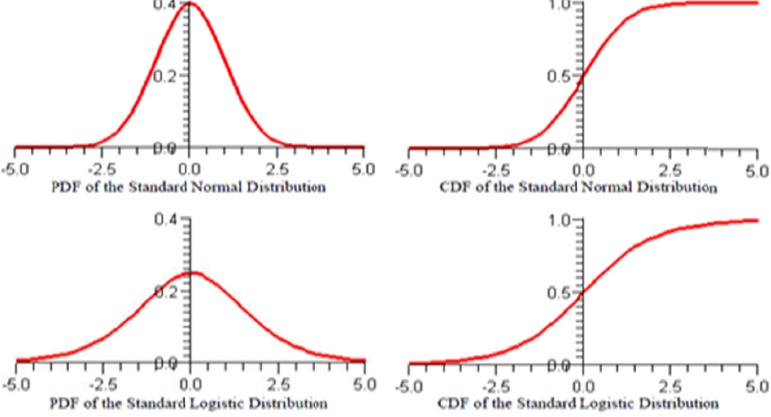
The standard normal and standard logistic probilitydistributons

The probability density function (PDF) of the standard normal probability distribution has ahigher peak and thinner tails than the standard logistic probability distribution (Figure 1). The standard logistic distribution looks as if someone has weighed down the peak of the standard normal distribution and strained its tails. As a result, the cumulative density function (CDF) of the standard normal distribution is steeper in the middle than the CDF of the standard logistic distribution and quickly approaches zero on the left and one on the right.

The two models, of course, produce different parameter estimates. In binary response models, the estimates of a Logit model are roughly 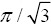 times larger than those of the probit model. These estimators, however, end up with almost the same standardized impacts of independent variables Scott Long (1997).

The choice between Logit and probit model is more closely related to estimation and familiarity rather than theoretical and interpretive aspects. In general, Logit models reach convergence fairly well. Although some (multinomial) probit models may take a long time to reach convergence, a probit model works well for bivariate models. As computing power improve sand new algorithms are developed, importance of this issue is diminishing. For discussion on choosing logit and probit models, see Cameron and Trivedi (2009, pp. 471-474):

In this study, the logarithm of the income variable is used. Because of the logarithmic form of income variable obeys normal distribution.

**Figure 2:**
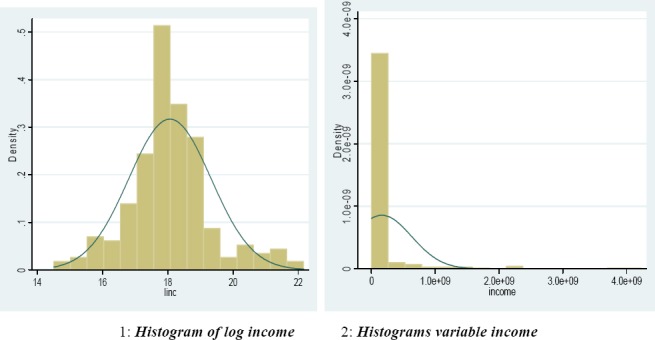


## 3. Findings

All variables were estimated in Logit and probitmodels (Appendix 1), and the significance level was determined, after consideration of factors such as the number of significant variables, the logarithm of the maximum likelihood, Akaike and Bayesian statistics, the probit model was chosen and themarginal effect for each of the significant variables including gender, occupation, education, income, deterioration, need, residential status and family size were calculated.

To analyze the data, using the default software STATA12, probit model was considered and the results were compared with the results of the Logit Model:

- Number of estimated significant variables are 8 significant variable in the probit model; and 6 variables in the Logit model is significant. Probit model is optimal in terms of number of significant variables.
- Based on Maximum likelihood logarithms: probit model (-14.58) and the Logit model with (-14.86), Probit model is much larger than the Logit one and the probit model is optimized.
- Based on Akaike statistic: the probit model Akaike criterion equals 59.16 vs. Logit models with Akaike criterion value 59.72. So the probit model is selected.
- Based on Bayesian statistics: the standard Bayesian probit model with a value of 110.405 compared to the standard Bayesian Logit model equals 110.964, the probit model is optimized.
- So Theprobit model was chosen and the marginal effects of the variables were calculated. To assess the overall significance of the regression we can use Wald statistics. Wald statistic equals 82.19 in our probit model which is greater than the critical value in chi-square distribution table (freedom degree 14 & significant level 0.01), so our estimated model is significant.

Pseudo coefficient equals 0.7 in estimated model which indicates that more than 70% of changes in dependent variable can be explained by independent variables of the model.

**Table 5:** Parameters of probit & Logit model

Parameters	Probit model	Logit model
The Wald statistic and degrees of freedom	82.19(14)	68.58(14)
Wald statistic Significance level	0.000	0.000
pseudo coefficient of determination	0.7065	0.7009
Logpseudolikelihood	-14.582	-14.86

Source: research findings.

**Table 6:** Results of the probit model

Significance level	Z statistics	Standard deviation	Coefficient	variables
0.631	0.48	0.537489	0.25852	Insurance
0.308	-1.02	0.014552	-0.01485	Age
0.06	1.88	0.16311	0.306322	Household size
0.00	6.79	0.687125	4.668486	Need
0.089	-1.7	0.628836	-1.07048	Deterioration
0.038	-2.07	0.764164	-1.58526	House Ownership
0.00	-3.97	0.475239	-1.88771	Sex
0.007	-2.7	0.182209	-0.49256	Log Income
Education				
0.052	-1.95	0.724011	-1.40827	High school
0.288	-1.06	0.776065	-0.82506	University
Kind of drugstore				
0.957	0.05	0.707773	0.038423	Charity
0.828	-0.22	0.395256	-0.08611	Governmental
Occupation				
0.085	-1.72	0.441299	-0.76046	Non Governmental
0.269	-1.11	0.676273	-0.74759	Jobless
0.005	2.83	3.850997	10.90508	Constant

**Table 7:** Final results of the probit model: Marginal effects and the significant level

variables	Coefficient	Standard deviation	Z statistics	Significance level	Marginal effect
Household size	0.306322	0.16311	1.88	0.06	0.000486
Need	4.668486	0.687125	6.79	0.000	0.858405
Deterioration	-1.07048	0.628836	-1.7	0.089	-0.00179
House Ownership	-1.58526	0.764164	-2.07	0.038	-0.00263
Sex	-1.88771	0.475239	-3.97	.000	-0.0479
Log Income	-0.49256	0.182209	-2.7	0.007	-0.00078
Education				
High school	-1.40827	0.724011	-1.95	0.052	-0.0021
University	-0.82506	0.776065	-1.06	0.288	-0.00335
Occupation				
Non Governmental	-0.76046	0.441299	-1.72	0.085	-0.00171
Jobless	-0.74759	0.676273	-1.11	0.269	-0.00272
Constant	10.90508	3.850997	2.83	0.005	0.000338

### 3.1 Hosmer-Lemeshow Statistics

Finally, to evaluate the model in terms of the presence or absence of issues such as: varianceheterscedasticity, autocorrelation, Hosmer-Lemeshow test was calculated. This statistic equals 0.54 which is smaller than the critical value of chi-square distribution table at the 1% significance level, 20.09 (with degrees of freedom equal to 8).

## 4. Conclusion

After estimating the different variables in the Logit and probit models and determine their significance level, and examines factors such as the number of significant variables, the logarithm of the maximum likelihood statistic, Akaike and Bayesian statistics, the probit model was selected. And Marginal effects for each of the significant variables were calculated. As mentioned above, the ability to pay for medications, especially in certain chronic diseases, and the use of mechanisms to protect the poor groups is vital.

Findings show that relationship between insurance coverage and drug demand is not statistically significant. This may due to several causes: First, the need for medication and treatment in these patients, who are suffering from certain expensive diseases, is critical and patients pay for it regardless of insurance coverage. Second can be inefficiencies due to the contribution and amount of insurance coverage for cost of medication. These findings are similar to Qiasvand (2009) that found the impact of health insurance program in Iran for the prevention of exposure to catastrophic health care expenditures has not been effective.

Household size has a positive and significant relationship with the demand for the drug demand. This relationship can be viewed from different perspectives; First, according to the study, which included patients with disease hemophilia and thalassemia, and due to hereditary and familial background of the diseases, there are likely to increase the number of children, number of sick family also increases. This leads to an increase in household demand. Another reason for the significant impact of household size on demand, may be creating a sense of responsibility toward patient member who required more care, this leads to greater demand for healthcare and drugs.

Deterioration in the general condition of patients have a significant negative effect on the health service is received. In this regard, it can be concluded that the person primarily due to lack of financial access, and low awareness, will be a face more serious condition. Secondly, the person with the worsening situation, due to a further increase costs and despair of recovery, less likely to try to meet his demand.

Sex of head of household has a negative and significant relationship with the demand for the service. The results show that household head men are less likely to take action to get the service. This result may be due to the active role of women in health; they do take most responsibility for the health of their family. Increasing levels of women’s education, training and enabling them may lead to remarkable achievements in improving health indicators and health promotion.

In the job category household working in the public sector is considered as the reference group. The results show that people with non-governmental job are less likely to take action to get the drug. It could be concluded that in private (non-governmental) jobs, there is less fixed income, which in turn will reduce the ability of households to finance drugs.

Three important variables remained include income, education and house ownership, it is better to discuss them together. Inverse significant correlation was observed in all three variables. In summary, increaseof the education level and income and house ownership decrease the demand for medication required. inthe study Rahbar et al. (2004), two models were estimated for urban and rural areas the income elasticity of demand, representing 0.45 percent increase in drug demand due to a unit change in income. This is against our findings.

If we assume these three variables as welfare components. According to previous studies, increase in each of them may lead to increased demand, but in our study which include MS and renal failure patients. It should be noted that MS patients strongly influenced by environmental factors, stress, family issues and concerns caused by poverty and the fear of failing to provide expensive drugs. These factors can have a significant impact on disease recurrence. MS patients with increased welfare component, such as income, housing and education, feel the relative tranquility and the relapse rate is less experienced so less demand for the drug. Of course to obtain specific income elasticity of MS patients requires special study of this disease. Renal failure and its association with diet are very important. An individual diet that is suffering from kidney failure must be performed by a registered dietitian. Obviously having higher levels of education and income, improve the patient’s diet condition. And enjoying a proper diet may reduce needfor difficult and costly procedure of dialysis.

Given the growing importance and role of drugs in the treatment of disease, as well as replacement of costly and sometimes unsafe procedures, review of policies in cultural, social and economic context may be required. Issues such as health insurance coverage, NGO development, training and awareness of the patients, eliminating geographical barriers to access to medicines, nutritional training, dietary requirements associated with these diseases, the correct use and rational drug prescribing, the allocation of subsidies to specific drugs, could be medicines policy matters.

## Appendix I

**Table:**

Probit model estimations						Logit model estimations					

Significance level	Z statistics	Standard deviation	Marginal effect	Coefficient	variable	Significance level	Z statistics	Standard deviation	Marginal effect	Coefficient	variable
0.63	0.48	0.54	0.000	0.26	insurance	0.79	0.27	1.54	0.001	0.42	insurance
0.31	-1.02	0.01	0.000	-0.01	age	0.43	-0.79	0.03	0.000	-0.03	age
0.06	1.88	0.16	0.000	0.31	Household size	0.10	1.65	0.33	0.001	0.54	Household size
0.00	6.79	0.69	0.858	4.67	need	0.00	5.22	1.70	0.876	8.86	need
0.09	-1.70	0.63	-0.002	-1.07	Deterioration	0.15	-1.44	1.42	-0.003	-2.05	Deterioration
0.04	-2.07	0.76	-0.003	-1.59	House Ownership	0.17	-1.38	2.21	-0.004	-3.04	House Ownership
0.00	-3.97	0.48	-0.048	-1.89	Sex	0.00	-3.39	1.14	-0.047	-3.85	Sex
0.05	-1.95	0.72	-0.002	-1.41	High school	0.03	-2.14	1.19	-0.004	-2.56	High school
0.29	-1.06	0.78	-0.003	-0.83	University	0.29	-1.06	1.36	-0.004	-1.44	University
0.96	0.05	0.71	0.000	0.04	Charities	0.82	0.23	1.64	0.001	0.38	Charities
0.83	-0.22	0.40	0.000	-0.09	Governmental	0.97	-0.03	0.86	0.000	-0.03	Governmental
0.01	-2.70	0.18	-0.001	-0.49	Log Income	0.02	-2.38	0.42	-0.002	-1.01	Log Income
0.09	-1.72	0.44	-0.002	-0.76	Private	0.12	-1.57	0.88	-0.003	-1.38	Private
0.27	-1.11	0.68	-0.003	-0.75	Jobless	0.42	-0.82	1.39	-0.003	-1.13	Jobless
0.01	2.83	3.85		10.91	Y	0.02	2.42	9.03		21.89	Y
